# Vapor and Liquid Phase Profiles of Essential Oils from *Abies*, *Picea* and *Pinus* Species and Their Phytotoxic Interactions with Weed Growth in Pre- and Post-Emergence Conditions

**DOI:** 10.3390/plants12051172

**Published:** 2023-03-03

**Authors:** Stefania Garzoli, Valentina Vaglia, Marcello Iriti, Sara Vitalini

**Affiliations:** 1 Department of Drug Chemistry and Technology, Sapienza University, P.le Aldo Moro 5, 00185 Rome, Italy; 2 Department of Environmental Science and Policy, Università degli Studi di Milano, Via G. Celoria 2, 20133 Milan, Italy; 3 Department of Biomedical, Surgical and Dental Sciences, Università degli Studi di Milano, Via G. Pascal 36, 20133 Milan, Italy; 4 National Interuniversity Consortium of Materials Science and Technology, Via G. Giusti 9, 50121 Firenze, Italy

**Keywords:** *Abies alba*, *Picea abies*, *Pinus cembra*, *Pinus mugo*, chemical volatile composition, SPME-GC-MS, allelopathy, weed management

## Abstract

The chemical content of essential oils (EO) obtained from the leaves of four Pinaceae (*Abies alba*, *Picea abies*, *Pinus cembra* and *Pinus mugo*) was investigated by SPME-GC-MS technique. The vapor phase was characterized by the monoterpenes with values higher than 95.0%. Among them, *α*-pinene (24.7–48.5%), limonene (17.2–33.1%) and *β*-myrcene (9.2–27.8%) were the most abundant. The monoterpenic fraction prevailed over the sesquiterpenic one (≥74.7%) in the EO liquid phase. Limonene was the major compound in *A. alba* (30.4%), *P. abies* (20.3%) and *P. mugo* (78.5%), while *α*-pinene in *P. cembra* (36.2%). Regarding the phytotoxic properties, EOs were studied at different doses (2–100 μL) and concentrations (2–20/100 μL/mL). All EOs were found to be significantly active (*p*-value ˂ 0.05) against the two recipient species in a dose-dependent way. In pre-emergence tests, germination of *Lolium multiflorum* and *Sinapis alba* was reduced by up to 62–66% and 65–82%, respectively, as well as their growth by up to 60–74% and 65–67%, due to the effects of compounds in both the vapor and liquid phases. In post-emergence conditions, at the highest concentration, the phytotoxicity of EOs caused heavy symptoms and, in the case of *S. alba*, *A. alba* EO completely destroyed (100%) the treated seedlings.

## 1. Introduction

Weeds are plants capable of causing qualitative and quantitative damage in terms of the yield and commercial value of agricultural products. These plants have characteristics that can increase the damage they cause, such as germination in adverse conditions, production of very long-lived and easily dispersible seeds, high competitiveness and rapid growth [1]. To have successful and sustainable crop production, it is necessary to develop alternative control strategies against weeds.

The chemical approach of using synthetic herbicides, often chosen for their speed and ease of use, is becoming increasingly problematic in terms of environmental pollution due to their low biodegradability and high persistence and percolation of the active compounds into soil and water [2]. Furthermore, due to the use of pesticides with similar mechanisms of action, resistance phenomena are also increasing [3]. Because of these issues, various formulations and active ingredients potentially capable of exerting an allelopathic action have been studied to be integrated with traditional weed control methods [4].

Allelochemicals must be released from the donor plant in a variety of ways, such as leaching from leaves and stems, volatilization from plant parts, and release by decomposition of organic residues or as root exudates. The phytotoxicity of these compounds are attributed to their ability to interfere with the biological mechanisms of the target plant at a biochemical (e.g., synthesis of DNA and protein alteration), physiological (e.g., respiration alteration) or structural (e.g., cuticle and cell membrane modification) level. The global effect of these mechanisms can be translated into reduced germination, a lower growth of shoots and roots, a lower absorption of nutrients and a decrease of the photosynthetic rate [5].

Essential oils (Eos) are among the products with allelopathic activity. When effective, their action causes rapid drying of the green parts of the plant by destroying the leaf cuticle and cell membranes [6]. EO-based herbicides have already been patented as natural and non-toxic products that can be used as a safe alternative for weed control in organic farming systems [7]. According to Verdeguer et al. [1], five commercial organic herbicides derived from EOs were available on the US market prior to 2020.

In this work, we focus on the phytotoxic potential of EOs obtained from four Pinaceae species, such as *Abies alba* Mill., *Picea abies* (L.) H.Karst., *Pinus cembra* L. and *Pinus mugo* Turra. To this end, *Lolium multiflorum* Lam. and *Sinapis alba* L., weeds of wheat in central-southern Italy and characterized by phenomena of resistance to conventional herbicides, including glyphosate [8,9], were used as receiver plants both in pre- and post-emergence tests. The volatile chemical profiles of the four EOs, both in vapor and liquid phase, were characterized by SPME-GC-MS and GC-MS techniques, respectively.

## 2. Results

### 2.1. Vapor Phase Chemical Composition of EOs

SPME-GC-MS analysis was performed to delineate the vapor phase profile of the EOs. In total, 55 compounds were identified, with 15 in *A. alba*, 18 in *P. abies*, 21 in *P. cembra* and 16 in *P. mugo* (Table 1). *α*-Pinene was the main compound in *A. alba* (48.5%) and *P. cembra* (52.9%), together with *β*-myrcene in *P. abies* (24.7% and 24.8%, respectively) and the second-most abundant component in *P. mugo* (28.0%), where limonene (33.1%) prevailed. Limonene was also detected at the same percentage (33.1%) in *A. alba* as well as in a slightly smaller amount in *P. cembra* (29.3%). *β*-myrcene was missing in *A. alba*, but significantly present in *P. cembra* (9.2%) and *P. mugo* (27.8%). The only ubiquitous sesquiterpene compound was *β*-caryophyllene which ranged from 0.1% to 0.4%. A series of compounds belonging to this chemical class were detected but with relative percentages lower than 0.1%. The exception was *δ*-cadinene which in *P. mugo* reached 0.3%.

### 2.2. Liquid Phase Chemical Composition of EOs

By gas chromatography–mass spectrometry (GC/MS) analysis, the liquid phase profile of EOs was described. A total of 69 compounds were identified, with 30 in *A. alba*, 45 in *P. abies*, 32 in *P. cembra* and 27 in *P. mugo* (Table 2). In all samples, the monoterpene fraction prevailed over the sesquiterpene fraction. Among the monoterpenes, limonene was the most abundant compound in *A. alba* (30.4%), *P. abies* (20.3%) and *P. mugo* (78.5%), while *α*-pinene in *P. cembra* (36.2%); however, significant differences in composition were found. For example, *β*-pinene (17.6%) and *β*-myrcene (11.3%) reached higher relative percentages in *P. abies* than in other EOs where they varied between 0.2% and 9.4%. Bornyl acetate, on the other hand, reached higher amounts in *A. alba* (7.3%) and *P. abies* (7.1%) than in *P. mugo* (1.7%) and *P. cembra* (1.5%). Among the sesquiterpenes, *β*-caryophyllene was the main compound in *A. alba* (9.6%), *P. abies* (3.7%) and *P. mugo* (2.0%) while *δ*-cadinene in *P. cembra* (2.4%).

### 2.3. Phytotoxicity of EOs in Vapor Phase

Table 3 shows the germination and growth values of *L. multiflorum* and *S. alba* whose seeds were treated (without direct contact) with four different doses of EOs. In general, both receiver species were significantly affected by the four EOs (*p*-value ˂ 0.05). *L. multiflorum* was more susceptible to the action of EO from *P. abies* and *A. alba*, *S. alba* to that of *P. cembra* and *P. mugo*. *P. abies* EO was more effective than *A. alba* and *P. cembra* than *P. mugo.*

Specifically, at the highest dose (100 μL), *P. abies* reduced germination of *L. multiflorum* by 62%, followed by *P. cembra* (−46%), *P. mugo* and *A. alba* (−39% and −38%, respectively). *P. abies* EO was the only one to significantly inhibit it (−57%), even at a dose of 50 μL. In contrast, all EOs were also significantly active at 2 and/or 20 μL against *S. alba*. Its germination decreased by 24–82% due to *P. cembra* (20–100 μL) and by 59–82% due to *P. mugo* (2–100 μL). *P. abies* (20–100 μL) diminished it by 24–60% while *A. alba* (2–100 μL) by 14–41%.

The GI, CVG and MGT indices confirmed this trend, highlighting the effectiveness of EOs in reducing germination speed and increasing germination times. *A. alba* and *P. abies* significantly decreased GI and CVG values of both *L. multiflorum* (−18% to −65% and −22% to −65%, respectively) and *S. alba* (−15% to −50% and −21% to −57%) starting from 2 μL. *P. cembra* and *P. mugo* were less effective against *L. multiflorum*, obtaining remarkable results only at 50 and 100 μL (GI, −53% to −62% and −27% to −53%; CVG, −58% to −66% and −33% to −57%). Both EOs were able to significantly increase *L. multiflorum* MGTs only at 100 μL (+10% and +15%, respectively).

Concerning seedling growth, EOs from *A. alba* and *P. abies,* at 100 μL, similarly reduced shoot elongation of *L. multiflorum* (by about 60%), while *P. cembra* inhibited it by 42% and *P. mugo* by 28%. Slightly higher percentages were reached in the developmental arrest of *S. alba*, even if by different EOs (*P. cembra* > *P. abies* > *P. mugo* > *A. alba*). All EOs were significantly active against both receiver species even at 50 µL (up to −55% for *L. multiflorum* and up to −42% for *S. alba*) while their lowest doses (2 and 20 μL) against *S. alba* only.

### 2.4. Phytotoxicity of EOs in Liquid Phase

Table 4 shows the germination and growth values of *L. multiflorum* and *S. alba* whose seeds were treated (direct contact) with four different concentrations of EOs. As in the previous test, both receiver species were significantly affected by the four EOs (*p*-value ˂ 0.05), albeit differently. Again, *L. multiflorum* was more sensitive to the activity of *A. alba* and *P. abies*, but in this case, *A. alba* had a stronger effect than *P. abies*. *S. alba* was mostly affected by *P. abies* and *P. mugo*, with some exceptions. In general, *A. alba* EO was more effective than *P. abies* and *P. cembra* than *P. mugo*.

In detail, *A. alba* was able to decrease the germination of *L. multiflorum* more than *P. abies*, *P. cembra* and *P. mugo* (up to −66%, −51%, −47% and −42%, respectively) while being active only at 20 and 10 μL/mL, unlike other EOs that were effective even at lower concentrations (2 and/or 5 μL/mL). In contrast, *A. alba* did not significantly inhibit *S. alba* germination, unlike *P. abies*, *P. cembra* and *P. mugo*, which reduced it by up to 65%, 43% and 55%, respectively.

The EO impact data on the germination of the receiver species were corroborated by the relative indices. At the four used concentrations, *A. alba* decreased GI and CVG values of *L. multiflorum* similarly (−28% to −84% and −27% to −85%, respectively), followed by *P. abies* (−22% to −73% and −25% to −73%), *P. cembra* (−53% to −71% and −55% to −71%) and *P. mugo* (−19% to −52% and −21% to −57%). In the case of *S. alba*, only *P. cembra* did not significantly change GIs and CVGs at 2 μL/mL. *P. abies* reduced them by 47–74% and 27–79%, *P. mugo* by 25–68% and 25–71%, *A. alba* by 14–61% and 13–47%. On the other hand, *L. multiflorum* and *S. alba* MGT values increased up to a maximum of 16% and 21%, respectively, due to *A. alba* and *P. abies* EOs. The least effective EOs were those from *P. abies* and *P. mugo*, which were effective on *L. multiflorum* only at the highest concentrations (in the first case, at 10 and 20 μL/mL with a 10% and 16% increase in MGT; in the second case, at 20 μL/mL with a 14% MGT increase).

As for seedling development, *A. alba*, despite being significantly active only at 20 μL/mL, was the EO most capable of stunting the growth of *L. multiflorum* by reducing shoot length by 74%. *P. cembra* achieved a slightly lower result (−67%), while *P. abies* and *P. mugo* showed comparable activity (−51% and −50%). Similar percentages were obtained for *S. alba*, but with different efficacy of the EOs (*P. cembra*, −67% > *P. abies*, −56% > *P. mugo*, −50% > *A. alba*, −48%).

### 2.5. Effectiveness of EOs in Post-Emergence Conditions

Table 5 shows the final effects (after 48 h) of EOs on *L. multiflorum* and *S. alba* seedlings at 10–100 μL/mL. Their lowest tested concentrations (2 and 5 μL/mL) achieved no effect. However, all EOs significantly damaged both receiver species starting from the 10 μL/mL concentration, affecting up to 28.9% of treated seedlings with a degree of toxicity of the considered scale (Table 6) equal to 1–2 (very slight to more severe, but not lasting symptoms).

At 20 µL/mL, the phytotoxicity of EOs towards *L. multiflorum* varied between 2 and 4 (medium and lasting symptoms), with damage to 42.5–55.6% seedlings, 33.6–41.1% leaves, and 24.8–32.7% of their leaf surfaces. At 50 μL/mL, *A. alba* and *P. mugo* were successful in causing moderately heavy to heavy damage to *L. multiflorum* with symptoms affecting more than 50% of the leaf area (68.8% and 58.8%, respectively), destroying it almost completely at 100 μL/mL (≥96% damaged leaves). *P. abies* produced moderate to very severe symptoms (100% damaged seedlings, ≥96.7% damaged leaves, ≥54.7% damaged leaf surface) while *P. cembra* provoked moderate and lasting to heavy symptoms (≥86.7% damaged seedlings, ≥76.7% damaged leaves, ≥59.1% damaged leaf surface).

*S. alba* was found to be even more susceptible than *L. multiflorum* to the action of EOs. At 50 and 100 μL/mL, *A. alba* destroyed its seedlings (100%), starting to hit them heavily at 20 μL/mL (69.4% damaged seedlings, 60.5% damaged leaves, 52.2% damaged leaf surface). At the highest concentration, *P. mugo* caused similar symptoms affecting 100% of seedlings and their leaves with more than 80% of the surface damaged. At 20 and 50 μL/mL, it caused medium and lasting to very heavy symptoms injuring 57.7–75.5% of seedlings. *P. abies* and *P. cembra* showed similar effects (51.1–100% and 55.6–100% damaged seedlings, 44.4–99.0% and 54.4–97% damaged leaves, 27.7–67.2% and 30.5–70.6% damaged leaf surface) with moderate and longer lasting to very severe symptoms.

Lastly, 24 h after the first treatment, only *A. alba* and *P. cembra* EOs were also significantly active at the concentration of 10 μL/mL against *S. alba* (data not shown).

## 3. Discussion

The chemical profile of four EOs from Pinaceae—*A. alba*, *P. abies*, *P. cembra* and *P. mugo*—tested for phytotoxic interactions with weeds was investigated by SPME-GC/MS. From a qualitative point of view, the EOs vapor phase was less rich in components than the respective liquid phase while, quantitatively, the percentage values of the more volatile compounds, i.e., the lower boiling temperature, prevailed. Nevertheless, in all samples, both phases were characterized by the prevalence of monoterpene compounds in accordance with the composition data of Pinaceae EOs [10,11,12,13,14,15].

A wide variety of activities, including allelopathic activity, are attributed to monoterpenes [16]. In particular, they have a marked synergistic phytotoxic action when used in combination, supporting a greater effect of crude EOs than single components [17,18,19], some of which, however, have shown a remarkable ability to suppress weed germination and growth. For example, Kordali et al. [20] demonstrated that most of the 30 investigated monoterpenes possessed significant inhibitory activity with species-specific effects and that the oxygenated ones were more active than the hydrocarbon ones. Among the most effective compounds and with comparable phytotoxicity were limonene, myrcene, *α*-pinene and *β*-pinene, the four main constituents of the EOs investigated herein.

The weed-suppressing potential of limonene was later also assessed by Vaid and co-authors [21], who discovered that even at low concentrations, it was able to inhibit germination and seedling growth in terms of height, dry weight and root elongation of *Amaranthus viridis* L. Zhao et al. [22] studied the impact of limonene on *Chlorella vulgaris* cell growth reporting that it could play allelopathic roles in cyanobacterial VOCs by reducing the photosynthetic capabilities of algae. Moreover, Jalaei and collaborators [23] identified limonene as the second most abundant compound in the EO of *Dracocephalum kotschyi* Boiss., capable of reducing seed germination and seedling growth of two important weeds such as *Amaranthus retroflexus* L. and *Chenopodium album* L. The phytotoxic activity of limonene and *β*-myrcene was further investigated, against both monocotyledonous and dicotyledonous weeds. The obtained data confirmed their potential as allelochemicals [17]. Pal Singh et al. [18] found *β*-myrcene as the most toxic constituent of *Artemisia scoparia* Waldst. & Kit. leaf EO, followed by *p*-cymene and limonene, suggesting that further exploration is warranted in terms of its phytotoxicity against weeds. On the other hand, Raha [24] showed that the germination of *Echinochloa crus-galli* L. was not significantly affected by exposure to limonene and *β*-myrcene, which, however, consistently and proportionally inhibited its root development to the used concentrations.

*β*-Myrcene was also identified as one of the major volatile constituents of *Artemisia frigida* Eichw. released from fresh leaves crushed and tested for their allelopathic activity with positive feedback [25]. Recently, Chen et al. [26] reported a strong phytotoxic activity of *α*-pinene against *Elymus nutans* Griseb., an important forage and ecological restoration plant species. This monoterpene was able to severely stress the target seedlings at very low concentrations. Different effects of pinene isomers against *Zea mays* L. were also documented by Areco et al. [27], observing a more incisive action of *β*-pinene compared to *α*-pinene. The same information was previously recorded by Nishida et al. [28]. They also verified that these monoterpenes may cause inhibition of cell proliferation in the root apical meristem of the test plants. Finally, high allelopathic activity was recorded for the EOs of *Prangos pabularia* Lindl. leaves, whose main compound was *α*-pinene [29].

As a whole, the results of this work confirmed the efficacy of Pinaceae EOs as well as their chemical content. For instance, the strong activity of EO from *Pinus halepensis* Mill. needles were attributed to the high content in monoterpenes, especially myrcene, but also *α*-pinene and *β*-pinene, by Aidi Wannes et al. [30] corroborating the hypothesis of Hamrouni and co-authors [31]. Likewise, the inhibiory effects of *Pinus taeda* L. EO on radicle elongation of *Lolium* species was referred to its major compounds such as *α*-pinene, *β*-pinene and limonene [32]. In contrast, for other EOs from Pinaceae leaves, including that of *A. alba*, our data refuted their inability to negatively affect the weed development [33], at least against *S. alba* and *L. multiflorum*.

## 4. Materials and Methods

### 4.1. Plant Material

Organic EOs from needle-like leaves of *A. alba*, *P. abies*, *P. cembra* and *P. mugo* (lots n. 112009, 100410, 111509 and 103006, respectively) were provided by the Bergila family business located in Falzes (Bolzano, Italy) and stored at 4 °C until use.

The target seeds of *L. multiflorum* and *S. alba* were respectively supplied by the organic farm “Terre di Lomellina” (Pavia, Italy) and by the company “Padana Sementi” (Padova, Italy).

### 4.2. Solid-Phase Microextraction (SPME)

To describe the chemical volatile profile of the EOs, a SPME device from Supelco (Bellefonte, PA, USA) was used for the sampling. The EOs (~0.5 mL) were individually placed into a 7 mL glass vial with PTFE-coated silicone septum. The chosen fiber was coated with 50/30 μm DVB/CAR/PDMS (divinylbenzene/carboxen/polydimethylsiloxane). Before sampling, the samples were equilibrated for 20 min at 50 °C. Subsequently, the fiber was exposed to the equilibrated headspace for 10 min to capture the components released from the EOs. Later, the fiber was inserted in the GC injector maintained at 250 °C for the desorption of collected components. Before each sampling, the fiber was regenerated at 270 °C for 20 min in the injector port.

### 4.3. Gas Chromatography/Mass Spectrometry (GC/MS)

A Clarus 500 model (Perkin Elmer, Waltham, MA, USA) gas chromatograph coupled with a mass spectrometer and equipped with a FID (flame detector ionization) was used to carry out the analyses. A Varian Factor Four VF-1 capillary column was used to obtain the separation of the components and helium was used as carrier gas at a constant flow of 1 mL/min. The adopted chromatographic conditions followed those noted in [34]. The mass spectra were obtained in the electron impact mode (EI) at 70 eV in scan mode in the range 35–400 *m*/*z*. The identification of the compounds was performed by matching their mass spectra with those stored in the Wiley 2.2 mass spectrum library database and by comparison of the calculated linear retention indices (LRIs), obtained using a mixture of C_8_–C_25_ *n*-alkanes analyzed under the same conditions, with those available in the literature. Relative amounts of the compounds (percentage values) were calculated in relation to the total area of the chromatogram by normalizing the peak area. No internal standard nor factor correction were used. All analyses were carried out in triplicate.

### 4.4. Phytotoxicity

#### 4.4.1. Pre-Emergence Test with EOs in Vapor Phase

Fifteen sterilized seeds (1% NaClO) of *L. multiflorum* or *S. alba* were sown in 25 g of soil (Vigorplant^®^ SER CA 98 V7, Vigorplant Italia Srl, Lodi, Italy) inside Petri dishes (90 mm Ø) and wetted with 15 mL of sterilized water after placing at the same depth an appropriate number of sterile disks (6 mm Ø) impregnated with different doses of EOs (2, 20, 50 or 100 μL). In the controls, the EOs were replaced with distilled water. Samples were prepared in a sterile hood with vertical laminar flow. Then, the Petri dishes initialed and sealed with a double layer of Parafilm^®^ were incubated for 16 h light and 8 h darkness at 23 and 18 °C, respectively, in a climatic chamber for one week. The experimental design included four doses of each EO or distilled water × two target species × three replicates × two runs.

#### 4.4.2. Pre-Emergence Test with EOs in Liquid Phase

Fifteen sterilized seeds (1% NaClO) of *L. multiflorum* or *S. alba* were sown in 25 g of soil (Vigorplant^®^ SER CA 98 V7) inside Petri dishes (9 cm Ø) and wetted with 15 mL of the solution prepared with different concentrations of each EO (2, 5, 10 and 20 μL/mL) and Tween^®^ 20 (0.2%) as surfactant. In the controls, the EOs were replaced by 0.2% Tween^®^ 20 solution. Samples were prepared in a sterile hood with vertical laminar flow. Then, the Petri dishes initialed and sealed with a double layer of Parafilm^®^ were incubated for 16 h of light and 8 h of darkness at 23 and 18 °C, respectively, in a climatic chamber for one week. The experimental design included 4 concentrations of each EO or distilled water × 2 target species × 3 replicates × 2 runs.

#### 4.4.3. Post-Emergence Test with EOs in Liquid Phase

Ninety seeds total of *L. multiflorum* or *S. alba*, after germination in Petri dishes (9 cm Ø) in a growth chamber at 25 °C/16 h of light and 18 °C/8 h of dark, were transferred into 6 pots (13 cm Ø) filled with soil (Vigorplant^®^ SER CA 98 V7). Three weeks later, the plants with fully expanded first leaves were exposed to the treatments by spraying them every 24 h twice with the solution prepared using different concentrations (2 to 100 μL/mL) of each EO plus Tween^®^ 20 (0.2%) as surfactant. Control plants were instead sprayed with 0.2% Tween^®^ 20 solution. In all cases, the leaves were covered with small droplets, up to just before the point of outflow. The experimental design included six concentrations of each EO or distilled water × two target species × six replicates × two runs.

### 4.5. Data Processing

#### 4.5.1. Phytotoxicity Indices

The effects of the four EOs on germination and development of the target species in pre-emergence conditions were described using some indices such as (i) Germination percentage (G) = (Germinated seed number)/(Seed total number) × 100; (ii) Germination Index (GI) = (7 × N1) + (6 × N2) + … + (1 × N15), where N1, N2 ... N15 is the number of germinated seeds (up to a maximum of 15) on the first, second and subsequent days until 7th day; the multipliers (e.g., 7, 6 … 1) are weights given to the days of the germination [35]; (iii) Coefficient of Velocity of Germination (CVG) = (N1 + N2 + ... + Ni)/100 × (N1T1 + ... + NiTi), where N is the number of seeds germinated every day; T is the number of days from seeding corresponding to N [36]; (iv) Mean Germination Time (MGT) = (∑D × Germinated seed number)/(∑Germinated seed number), where D is the number of days from the beginning of germination, plus the number of seeds germinated on day D [37]. The data used for their calculation were obtained after sowing, monitoring germination every day for one week and measuring the length of seedling roots and shoots on the seventh day.

The effects of the EOs on the target species in post-emergence conditions were evaluated using the scale reported in Table 6 according to Nalini and Parthasarathi [38].

#### 4.5.2. Statistical Analysis

Data were assessed in IBM SPSS software by analysis of variance calculated separately for each EOs (i.e., *Abies alba*, *Picea abies*, *Pinus cembra* and *Pinus mugo*). All considered parameters (i.e., G%, GI, CVG, MGT, SVI, shoot length) for the two receiver species (i.e., *L. multiflorum* and *S. alba*) under different treatments were taken into account as dependent variables.

The one-way ANOVA and the Tukey’s-b post-hoc test were used to determine the significant action (at *p* ≤ 0.05) of the EO treatments (i.e., the different EO doses or concentrations) on the receiver species and interpret the homogenous subsets.

## 5. Conclusions

The use of synthetic herbicides causes adverse effects on the environment and natural resources and human health. The need to identify new weed management approaches for incorporation into the increasingly sustainable development of agricultural practices has encouraged researchers to discover new potential active substances or mixtures. Based on the achieved results with *A. alba*, *P. abies*, *P. cembra* and *P. mugo*, it can be concluded that Pinaceae EOs are certainly an important source of active metabolites, in particular monoterpenes, whose potential can be exploited in the design of suitable bioherbicides, both of pre- and post-emergence, after carefully studying the involved molecular mechanism and evaluating aspects such as production costs, applicability and safety.

## Figures and Tables

**Table 1:** Vapor phase chemical composition (%) of *A. alba, P. abies*, *P. cembra* and *P. mugo* EOs, as determined by SPME-GC-MS.

N°	Component ^1^	Symbol ^2^ Class	LRI ^3^	LRI ^4^	*A. alba* (%) ^5^	*P. abies* (%) ^6^	*P. cembra* (%) ^7^	*P. mugo* (%) ^8^
1	santene	other	880	887	2.9 ± 0.02	1.0 ± 0.01	tr	-
2	cyclofenchene	m	892	896	tr	2.8 ± 0.03	tr	2.3 ± 0.02
3	tricyclene	m	913	920	2.1 ± 0.02	0.7 ± 0.02	1.0 ± 0.01	1.2 ± 0.01
4	*α*-pinene	m	938	943	48.5 ± 0.04	24.7 ± 0.06	52.9 ± 0.07	28.0 ± 0.04
5	camphene	m	940	946	1.8 ± 0.01	8.6 ± 0.02	2.0 ± 0.02	2.9 ± 0.02
6	sabinene	m	975	972	-	-	0.1 ± 0.01	0.1 ± 0.01
7	*β*-pinene	m	981	978	9.0 ± 0.03	16.5 ± 0.03	3.0 ± 0.02	-
8	*β*-myrcene	m	983	987	-	24.8 ± 0.05	9.2 ± 0.03	27.8 ± 0.05
9	*α*-phellandrene	m	1000	1005	-	-	0.2 ± 0.02	0.5 ± 0.01
10	limonene	m	1025	1023	33.1 ± 0.05	17.2 ± 0.04	29.3 ± 0.04	33.1 ± 0.06
11	trans-sabinene hydrate	m	1045	1052	-	-	tr	-
12	*γ*-terpinine	m	1057	1054	-	0.1 ± 0.01	tr	0.3 ± 0.02
13	terpinolene	m	1083	1080	0.2 ± 0.01	0.4 ± 0.01	0.2 ± 0.02	1.8 ± 0.02
14	*p*-cymenene	m	1092	1091	-	-	tr	-
15	fenchol	m	1094	1098	-	tr	-	-
16	L-pincarveol	m	1121	1119	-	-	0.1 ± 0.02	-
17	*α*-campholenal	m	1124	1125	-	-	tr	-
18	camphor	m	1130	1126	-	0.6 ± 0.02	-	-
19	sabina-ketone	m	1133	1132	-	-	0.1 ± 0.01	-
20	*cis*-sabinol	m	1135	1133	-	-	-	0.1 ± 0.01
21	pinocamphone	m	1146	1141	-	-	tr	-
22	camphene hydrate	m	1154	1149	-	0.2 ± 0.01	-	-
23	endo-borneol	m	1160	1155	0.1 ± 0.01	0.5 ± 0.01	0.1 ± 0.01	tr
24	terpinene-4-ol	m	1162	1160	-	0.1 ± 0.01	-	0.2 ± 0.01
25	*α*-terpineol	m	1188	1183	0.1 ± 0.01	0.2 ± 0.01	0.1 ± 0.01	0.1 ± 0.01
26	levoverbenone	m	1184	1191	-	tr	-	-
27	2-pinen-10-ol	m	1199	1194	-	-	tr	-
28	myrtenol	m	1200	1202	-	tr	-	-
29	methyl thymyl ether	m	1238	1234	-	tr	tr	0.2 ± 0.01
30	piperitone	m	1260	1254	-	tr	tr	-
31	bornyl acetate	m	1297	1290	1.3 ± 0.02	1.1 ± 0.03	0.2 ± 0.01	0.9 ± 0.02
32	*α*-terpinyl acetate	m	1350	1344	tr	-	0.1 ± 0.01	tr
33	citronellol acetate	m	1355	1348	0.1 ± 0.01	-	-	-
34	nerol acetate	m	1367	1363	tr	-	-	-
35	*α*-copaene	s	1377	1368	tr	-	tr	-
36	*α*-cubebene	s	1386	1381	tr	-	-	-
37	longicyclene	s	1400	1392	-	tr	-	-
38	α-longipinene	s	1406	1400	0.1 ± 0.01	tr	-	-
39	longifolene	s	1413	1408	0.1 ± 0.01	0.1 ± 0.01	-	-
40	*β*-caryophyllene	s	1429	1424	0.4 ± 0.02	0.1 ± 0.01	0.1 ± 0.01	0.3 ± 0.02
41	aromadendrene	s	1458	1460	-	-	tr	-
42	cis-muurola-4(15), 5-diene	s	1465	1461	-	tr	tr	tr
43	humulene	s	1471	1465	0.1 ± 0.01	tr	-	tr
44	cis-muurola-4(14), 5-diene	s	1483	1478	-	tr	-	tr
45	*γ*-muurolene	s	1494	1487	-	-	0.1 ± 0.01	-
46	*α*-franesene	s	1501	1496	tr	-	-	-
47	*β*-himachalene	s	1505	1495	tr	-	-	-
48	germacrene D	s	1509	1500	-	-	0.1 ± 0.01	-
49	*β*-bisabolene	s	1512	1501	-	-	0.1 ± 0.01	-
50	*δ*-cadinene	s	1524	1530	-	-	0.3 ± 0.02	tr
51	*α*-muurolene	s	1530	1534	-	-	0.1 ± 0.01	-
52	spathulenol	s	1612	1601	-	-	tr	-
53	*α*-bisabolol	s	1672	1668	-	-	tr	-
54	*α*-cadinol	s	1680	1676	-	-	tr	-
	SUM				99.9	99.7	99.4	99.8
	Monoterpenes				96.3	95.5	98.6	99.5
	Sesquiterpenes				0.7	0.2	0.8	0.3
	Other				2.9	1.0	-	-

^1^ The components are reported according to their elution order on the apolar column. ^2^ Symbol for class compound, m—monoterpenes; s—sesquiterpenes. ^3^ Linear Retention indices measured on apolar column. ^4^ Linear Retention indices from literature. ^5^ Percentage values of *A. alba* EO components (%). ^6^ Percentage values of *P. abies* EO components. ^7^ Percentage mean values of *P. cembra* EO components. ^8^ Percentage mean values of *P. mugo* EO components. -: Not detected; tr: traces (mean value < 0.1%).

**Table 2:** Liquid phase chemical composition (%) of *A. alba*, *P. abies*, *P. cembra* and *P. mugo* EOs, as determined by GC-MS.

N°	Component ^1^	Symbol ^2^ Class	LRI ^3^	LRI ^4^	*A. alba* (%) ^5^	*P. abies* (%) ^6^	*P. cembra* (%) ^7^	*P. mugo* (%) ^8^
1	santene	other	880	887	0.8 ± 0.02	0.3 ± 0.02	-	-
2	cyclofenchene	m	892	896	0.4 ± 0.02	2.5 ± 0.02	1.0 ± 0.02	4.4 ± 0.03
3	tricyclene	m	913	920	1.0 ± 0.03	0.4 ± 0.02	0.1 ± 0.02	-
4	*α*-pinene	m	938	943	20.6 ± 0.06	12.4 ± 0.03	36.2 ± 0.06	3.5 ± 0.02
5	camphene	m	940	946	8.1 ± 0.02	4.9 ± 0.02	1.5 ± 0.02	0.4 ± 0.02
6	sabinene	m	975	972	-	0.2 ± 0.01	0.1 ± 0.01	-
7	*β*-pinene	m	981	978	4.9 ± 0.02	17.6 ± 0.03	1.3 ± 0.02	0.2 ± 0.02
8	*β*-myrcene	m	983	987	tr	11.3 ± 0.03	9.4 ± 0.04	5.1 ± 0.03
9	*α*-phellandrene	m	1000	1005	-	-	-	0.1 ± 0.02
10	*p*-cymene	m	1010	1016	-	0.3 ± 0.02	-	-
11	limonene	m	1025	1023	30.4 ± 0.04	20.3 ± 0.05	34.3 ± 0.05	78.5 ± 0.08
12	*γ*-terpinene	m	1057	1054	-	0.2 ± 0.02	0.1 ± 0.01	0.1 ± 0.01
13	terpinolene	m	1083	1080	0.3 ± 0.02	0.8 ± 0.02	0.4 ± 0.02	0.6 ± 0.02
14	linalool	m	1087	1095	0.2 ± 0.02	-	-	-
15	*p*-cymenene	m	1092	1091	-	-	-	0.1 ± 0.01
16	fenchol	m	1094	1098	-	0.1 ± 0.01	-	-
17	L-pincarveol	m	1121	1119	-	-	2.3 ± 0.02	-
18	*α*-campholenal	m	1124	1125	0.3 ± 0.02	-	tr	-
19	camphor	m	1130	1126	-	1.6 ± 0.03	-	0.1 ± 0.02
20	*cis*-sabinol	m	1135	1133	tr	-	-	-
21	camphene hydrate	m	1154	1149	-	0.6 ± 0.02	-	-
22	endo-borneol	m	1160	1155	tr	1.9 ± 0.02	0.1 ± 0.01	0.1 ± 0.01
23	terpinene-4-ol	m	1162	1160	-	0.4 ± 0.02		0.3 ± 0.02
24	isothujol	m	1169	1165	0.3 ± 0.02	-		
25	*α*-terpineol	m	1188	1183	0.1 ± 0.01	0.9 ± 0.02	0.5 ± 0.02	0.1 ± 0.02
26	crypton	m	1192	1188	-	-	0.4 ± 0.02	-
27	2-pinen-10-ol	m	1199	1194	-	0.1 ± 0.02	-	-
28	linalyl formate	m	1205	1206	0.3 ± 0.02	-	-	-
29	citronellol	m	1219	1212	-	0.1 ± 0.01	-	0.1 ± 0.01
30	methyl thymyl ether	m	1238	1234	-	0.2 ± 0.02	0.6 ± 0.02	0.2 ± 0.02
31	isobornyl formate	m	1240	1237	-	0.1 ± 0.01	-	-
32	*α*-ocimene	m	1244	1239	-	0.1 ± 0.01	-	-
33	piperitone	m	1260	1254	-	0.1 ± 0.01	-	-
34	2-undecanone	m	1281	1276	-	0.1 ± 0.01	-	-
35	bornyl acetate	m	1297	1290	7.3 ± 0.03	7.1 ± 0.03	1.5 ± 0.02	1.7 ± 0.02
36	*α*-terpinyl acetate	m	1350	1344	-	0.5 ± 0.02	0.5 ± 0.02	0.3 ± 0.02
37	citronellol acetate	m	1355	1348	0.8 ± 0.02	0.1 ± 0.02	-	-
38	nerol acetate	m	1367	1363	0.6 ± 0.02	-	-	-
39	*α*-copaene	s	1377	1368	-	-	0.1 ± 0.01	-
40	*α*-cubebene	s	1386	1381	0.2 ± 0.02	-	0.1 ± 0.00	-
41	longicyclene	s	1400	1392	-	0.4 ± 0.02	-	-
42	α-longipinene	s	1406	1400	1.8 ± 0.02	-	-	-
43	longifolene	s	1413	1408	1.1 ± 0.02	2.7 ± 0.03	-	-
44	*β*-caryophyllene	s	1429	1424	9.6 ± 0.04	3.7 ± 0.03	0.9 ± 0.03	2.0 ± 0.02
45	aromadendrene	s	1458	1460	1.5 ± 0.02	-	0.2 ± 0.02	-
46	humulene	s	1471	1465	3.4 ± 0.02	1.4 ± 0.02	0.5 ± 0.02	0.3 ± 0.02
47	*β*-eudesmene	s	1490	1483	0.4 ± 0.02	-	-	-
48	*γ*-muurolene	s	1494	1487	-	0.2 ± 0.02	0.9 ± 0.02	0.1 ± 0.01
49	*β*-bisabolene	s	1499	1494	0.9 ± 0.02	-	-	-
50	*α*-franesene	s	1501	1496	-	0.3 ± 0.02	-	-
51	*β*-himachalene	s	1505	1495	0.9 ± 0.02	-	-	-
52	germacrene D	s	1509	1500	-	0.5 ± 0.02	1.5 ± 0.02	0.2 ± 0.02
53	*β*-bisabolene	s	1512	1501	-	0.3 ± 0.02	0.7 ± 0.02	-
54	*γ*-cadinene	s	1515	1508	-	-	0.9 ± 0.02	-
55	*δ*-cadinene	s	1524	1530	1.8 ± 0.02	1.9 ± 0.03	2.4 ± 0.02	0.5 ± 0.02
56	*α*-muurolene	s	1530	1534	-	0.5 ± 0.02	0.2 ± 0.02	0.3 ± 0.02
57	*α*-calacorene	s	1543	1539	0.2 ± 0.02	-	-	-
58	*β*-calacorene	s	1554	1548	tr	-	-	-
59	spathulenol	s	1612	1601	-	-	0.4 ± 0.02	0.1 ± 0.02
60	caryophyllene oxide	s	1617	1613	1.0 ± 0.01	0.2 ± 0.02	0.1 ± 0.00	0.2 ± 0.02
61	epicubenol	s	1622	1618	0.4 ± 0.02	0.2 ± 0.02	-	-
62	*β*-himachalol	s	1642	1637	0.7 ± 0.02	-	-	-
63	*τ*-muurolol	s	1653	1647	-	0.6 ± 0.02	-	-
64	*α*-bisabolol	s	1672	1668	-	tr	0.1 ± 0.01	-
65	*α*-cadinol	s	1680	1676	-	0.3 ± 0.02	0.2 ± 0.02	0.1 ± 0.00
66	cembrene	s	1955	1948	-	1.1 ± 0.02	-	-
67	*α*-camphorene	s	1976	1970	-	0.2 ± 0.02	-	-
68	geranyllinalool	s	2025	2020	-	0.2 ± 0.02	-	-
	SUM				99.4	99.9	96.4	99.7
	Monoterpenes				74.7	83.8	87.2	95.9
	Sesquiterpenes				23.9	15.7	9.2	3.8
	Other				0.8	0.4	-	-

^1^ The components are reported according to their elution order on apolar column; ^2^ Symbol for class compound, m—monoterpenes. s—sesquiterpenes. ^3^ Linear Retention indices measured on apolar column. ^4^ Linear Retention indices from literature. ^5^ Percentage values of *A. alba* EO components (%). ^6^ Percentage values of *P. abies* EO components. ^7^ Percentage mean values of *P. cembra* EO components (%). ^8^ Percentage mean values of *P. mugo* EO components. -: Not detected; tr: traces (mean value <0.1%).

**Table 3:** Germination and growth values of two receiver species under the phytotoxic effects of different doses of EOs (vapor phase) in pre-emergence conditions.

Target Species	EO (μL)	G (%)	GI	CVG	MGT	Shoot (mm)
	***A. alba***					
*L. multiflorum*	0	85 ± 8.5 a	158 ± 15.9 a	82 ± 11.3 a	5.2 ± 0.0 a	67 ± 2.4 a
	2	80 ± 5.7 a	130 ± 11.0 b	64 ± 7.6 b	5.2 ± 0.1 a	64 ± 3.5 a
	20	75 ± 10.0 a	114 ± 15.6 bc	62 ± 7.7 b	5.4 ± 0.1 ab	61 ± 4.2 a
	50	70 ± 11.5 a	87 ± 20.2 c	48 ± 13.5 b	5.6 ± 0.1 bc	50 ± 1.6 b
	100	53 ± 5.3 b	56 ± 8.8 d	29 ± 2.7 c	5.8 ± 0.2 c	26 ± 3.9 c
	*p*-value	0.001 *	0.000 *	0.000 *	0.007 *	0.000 *
*S. alba*	0	90 ± 11.5 a	247 ± 24.6 a	106 ± 15.6 a	4.3 ± 0.3 a	28 ± 1.1 a
	2	77 ± 4.0 b	209 ± 11.2 b	84 ± 5.9 b	4.6 ± 0.1 ab	25 ± 2.6 b
	20	77 ± 4.0 b	196 ± 10.4 b	82 ± 7.0 b	4.7 ± 0.1 b	24 ± 0.5 b
	50	67 ± 5.3 b	149 ± 23.6 c	57 ± 11.3 c	4.7 ± 0.1 b	19 ± 2.1 c
	100	53 ± 5.3 c	123 ± 19.8 c	46 ± 8.8 c	4.9 ± 0.1 b	18 ± 1.3 c
	*p*-value	0.000 *	0.000 *	0.000 *	0.007 *	0.000 *
	***P. abies***					
*L. multiflorum*	0	92 ± 6.2 a	179 ± 22.5 a	89 ± 5.7 a	5.0 ± 0.2 a	71 ± 3.1 a
	2	82 ± 6.7 ab	138 ± 9.5 b	70 ± 6.6 b	5.2 ± 0.0 ab	67 ± 5.7 a
	20	77 ± 11.6 ab	132 ± 13.5 b	66 ± 7.1 b	5.2 ± 0.0 ab	63 ± 4.6 a
	50	69 ± 8.3 b	103 ± 11.9 c	50 ± 3.9 c	5.3 ± 0.0 b	32 ± 3.5 b
	100	50 ± 3.5 c	33 ± 4.5 d	18 ± 3.8 d	6.1 ± 0.1 c	29 ± 2.6 b
	*p*-value	0.000 *	0.000 *	0.000 *	0.000 *	0.000 *
*S. alba*	0	88 ± 9.9 a	234 ± 18.8 a	101 ± 14.1 a	4.6 ± 0.1 a	33 ± 0.9 a
	2	78 ± 6.7 ab	205 ± 18.5 b	78 ± 7.4 b	4.8 ± 0.2 bc	29 ± 2.1 b
	20	67 ± 9.4 b	138 ± 17.6 c	60 ± 7.4 c	4.8 ± 0.1 bc	25 ± 2.8 c
	50	40 ± 13.5 c	84 ± 5.0 d	29 ± 7.5 d	5.0 ± 0.1 c	19 ± 2.5 d
	100	35 ± 4.4 c	82 ± 13.3 d	28 ± 1.6 d	5.0 ± 0.1 c	12 ± 1.4 e
	*p*-value	0.000 *	0.000 *	0.000 *	0.012 *	0.000 *
	***P. cembra***					
*L. multiflorum*	0	87 ± 7.5 a	173 ± 14.6 a	85 ± 10.2 a	5.1 ± 0.0 a	67 ± 3.9 a
	2	77 ± 4.0 ab	155 ± 7.5 a	73 ± 5.8 a	5.1 ± 0.0 a	58 ± 4.7 ab
	20	62 ± 15.6 bc	95 ± 20.6 b	44 ± 13.5 b	5.3 ± 0.1 b	50 ± 11.2 bc
	50	55 ± 10.0 bc	81 ± 12.8 b	36 ± 8.6 b	5.4 ± 0.1 b	45 ± 2.3 c
	100	47 ± 5.3 c	65 ± 16.2 b	29 ± 7.6 b	5.6 ± 0.1 c	39 ± 4.9 c
	*p*-value	0.000 *	0.000 *	0.000 *	0.012 *	0.000 *
*S. alba*	0	92 ± 8.3 a	257 ± 8.5 a	112 ± 14.1 a	4.6 ± 0.1 a	26 ± 0.7 a
	2	82 ± 8.3 ab	163 ± 20.1 b	77 ± 9.5 b	4.8 ± 0.1 b	21 ± 1.7 b
	20	70 ± 11.5 b	140 ± 12.8 b	65 ± 11.8 b	5.0 ± 0.1 b	17 ± 2.5 c
	50	27 ± 5.3 c	40 ± 8.1 c	14 ± 3.5 c	5.0 ± 0.1 b	16 ± 2.2 c
	100	17 ± 8.7 c	38 ± 14.1 c	12 ± 5.7 c	5.3 ± 0.1 c	9 ± 0.9 d
	*p*-value	0.000 *	0.000 *	0.000 *	0.000 *	0.000 *
	***P. mugo***					
*L. multiflorum*	0	82 ± 3.5 a	140 ± 7.4 a	72 ± 1.9 a	5.3 ± 0.1 a	69 ± 5.8 a
	2	75 ± 16.1 a	124 ± 11.4 ab	61 ± 12.0 ab	5.3 ± 0.0 a	62 ± 6.0 ab
	20	73 ± 5.3 a	120 ± 11.8 ab	59 ± 5.4 ab	5.3 ± 0.1 a	58 ± 4.1 bc
	50	64 ± 7.0 ab	102 ± 13.6 b	48 ± 8.2 b	5.4 ± 0.1 a	58 ± 2.7 bc
	100	50 ± 12.9 b	66 ± 10.5 c	31 ± 7.5 c	5.6 ± 0.1 b	50 ± 1.5 c
	*p*-value	0.005 *	0.000 *	0.000 *	0.000 *	0.000 *
*S. alba*	0	92 ± 8.3 a	252 ± 17.1 a	111 ± 12.2 a	4.6 ± 0.1 a	27 ± 1.8 a
	2	38 ± 6.7 b	67 ± 8.6 b	26 ± 4.4 b	4.8 ± 0.2 b	25 ± 2.3 ab
	20	33 ± 5.3 b	58 ± 11.7 b	22 ± 5.0 b	5.2 ± 0.1 c	21 ± 2.8 bc
	50	30 ± 6.5 b	54 ± 12.1 b	20 ± 5.7 b	5.3 ± 0.1 c	21 ± 0.3 c
	100	17 ± 4.0 c	42 ± 11.7 b	12 ± 3.5 b	5.3 ± 0.1 c	14 ± 2.7 d
	*p*-value	0.000 *	0.000 *	0.000 *	0.000 *	0.000 *

G%—Germination percentage; GI—Germination Index; CVG—Coefficient of Velocity of Germination; MGT—Mean Germination Time Values. Values are mean ± standard deviation; asterisk and different letters indicate statistically significant differences at *p*-value ≤ 0.05 among treatments in each species. *p*-value from ANOVA test.

**Table 4:** Germination and growth values of two target species under the phytotoxic effects of different concentrations of EOs (liquid phase) in pre-emergence conditions.

Target Species	EO (μL/mL)	G (%)	GI	CVG	MGT	Shoot (mm)
	***A. alba***					
*L. multiflorum*	0	87 ± 0.0 a	161 ± 13.7 a	80 ± 5.6 a	5.1 ± 0.1 a	65 ± 3.5 a
	2	82 ± 6.7 ab	116 ± 27.5 b	62 ± 11.7 b	5.4 ± 0.1 b	69 ± 5.1 a
	5	75 ± 11.5 ab	98 ± 17.7 bc	48 ± 12.3 b	5.5 ± 0.2 b	62 ± 12.6 a
	10	70 ± 11.6 b	80 ± 12.5 c	47 ± 9.9 b	5.8 ± 0.1 c	58 ± 5.7 a
	20	30 ± 3.5 c	26 ± 2.9 d	12 ± 1.9 c	5.9 ± 0.1 d	17 ± 2.2 b
	*p*-value	0.000 *	0.000 *	0.000 *	0.000 *	0.000 *
*S. alba*	0	85 ± 6.3 a	214 ± 30.5 a	94 ± 9.6 a	4.7 ± 0.1 a	29 ± 1.5 a
	2	80 ± 9.4 a	183 ± 11.4 b	82 ± 3.5 b	4.9 ± 0.1 b	18 ± 2.6 b
	5	80 ± 5.7 a	141 ± 4.9 c	67 ± 3.6 c	5.1 ± 0.1 c	18 ± 0.9 b
	10	78 ± 3.5 a	120 ± 9.0 c	58 ± 2.5 cd	5.2 ± 0.1 c	16 ± 0.9 b
	20	72 ± 8.3 a	84 ± 7.2 d	50 ± 4.2 d	5.7 ± 0.1 d	15 ± 1.5 b
	*p*-value	0.152	0.000 *	0.000 *	0.000 *	0.000 *
	***P. abies***					
*L. multiflorum*	0	90 ± 6.5 a	192 ± 22.6 a	92 ± 14.1 a	5.0 ± 0.0 a	64 ± 3.1 a
	2	78 ± 3.5 ab	149 ± 18.8 b	69 ± 9.6 b	5.0 ± 0.0 a	56 ± 1.4 b
	5	69 ± 8.3 b	121 ± 11.2 b	54 ± 7.6 b	5.1 ± 0.0 a	48 ± 4.6 c
	10	47 ± 7.4 c	67 ± 23.1 c	31 ± 10.0 c	5.5 ± 0.2 b	39 ± 3.9 d
	20	44 ± 4.0 c	51 ± 2.2 c	25 ± 1.3 c	5.8 ± 0.1 c	30 ± 2.6 e
	*p*-value	0.000 *	0.000 *	0.000 *	0.000 *	0.000 *
*S. alba*	0	82 ± 6.7 a	223 ± 11.6 a	94 ± 8.5 a	4.7 ± 0.1 a	27 ± 0.5 a
	2	63 ± 6.5 b	119 ± 17.4 b	69 ± 8.4 b	5.0 ± 0.1 b	19 ± 3.7 b
	5	55 ± 9.9 b	87 ± 12.2 c	48 ± 10.1 c	5.0 ± 0.1 b	16 ± 2.0 bc
	10	42 ± 3.5 c	69 ± 8.4 cd	33 ± 4.6 d	5.3 ± 0.1 c	13 ± 1.2 d
	20	29 ± 3.0 d	58 ± 10.1 d	20 ± 3.3 d	5.8 ± 0.1 d	12 ± 3.2 d
	*p*-value	0.000 *	0.000 *	0.000 *	0.000 *	0.000 *
	***P. cembra***					
*L. multiflorum*	0	100 ± 0.0 a	190 ± 14.7 a	99 ± 7.2 a	5.1 ± 0.1 a	72 ± 3.9 a
	2	75 ± 14.6 b	90 ± 13.8 b	45 ± 9.3 b	5.5 ± 0.0 b	63 ± 2.1 b
	5	67 ± 9.4 bc	84 ± 12.0 b	42 ± 8.8 bc	5.5 ± 0.1 b	53 ± 2.7 c
	10	58 ± 3.5 c	82 ± 5.8 b	38 ± 2.3 bc	5.5 ± 0.1 b	35 ± 3.7 d
	20	53 ± 0.0 c	55 ± 2.6 c	29 ± 1.7 d	5.8 ± 0.1 c	24 ± 2.2 e
	*p*-value	0.000 *	0.000 *	0.000 *	0.000 *	0.000 *
*S. alba*	0	84 ± 4.0 a	218 ± 18.9 a	92 ± 8.5 a	4.7 ± 0.0 a	27 ± 1.8 a
	2	77 ± 15.8 a	184 ± 27.1 ab	80 ± 19.7 ab	4.8 ± 0.1 ab	27 ± 1.3 a
	5	69 ± 3.0 ab	151 ± 16.9 b	63 ± 8.2 bc	4.9 ± 0.1 ab	19 ± 1.2 b
	10	60 ± 0.0 bc	106 ± 21.0 c	44 ± 6.7 cd	5.1 ± 0.2 b	16 ± 2.1 c
	20	48 ± 6.2 c	97 ± 6.8 c	38 ± 3.4 d	5.1 ± 0.1 b	9 ± 1.4 d
	*p*-value	0.000 *	0.000 *	0.000 *	0.002 *	0.000 *
	***P. mugo***					
*L. multiflorum*	0	89 ± 3.0 a	190 ± 10.5 a	89 ± 7.1 a	4.9 ± 0.1 a	67 ± 2.3 a
	2	82 ± 8.0 a	153 ± 24.0 b	70 ± 13.0 b	5.0 ± 0.1 a	59 ± 4.0 b
	5	64 ± 4.0 b	118 ± 9.0 c	51 ± 4.0 c	5.1 ± 0.1 a	56 ± 2.0 b
	10	62 ± 3.5 b	95 ± 17.0 c	46 ± 5.0 c	5.2 ± 0.2 a	49 ± 4.0 c
	20	52 ± 6.0 c	91 ± 5.0 c	38 ± 5.0 c	5.6 ± 0.1 b	45 ± 3.0 c
	*p*-value	0.000 *	0.000 *	0.000 *	0.002 *	0.000 *
*S. alba*	0	89 ± 3.0 a	243 ± 10.6 a	105 ± 4.8 a	4.7 ± 0.1 a	28 ± 1.2 a
	2	72 ± 11.0 b	158 ± 23.0 b	68 ± 12.0 b	5.0 ± 0.0 b	24 ± 1.0 b
	5	65 ± 6.0 bc	145 ± 13.0 bc	60 ± 7.0 bc	5.0 ± 0.1 b	19 ± 1.0 c
	10	53 ± 5.0 c	121 ± 8.0 c	48 ± 3.0 c	5.5 ± 0.0 c	17 ± 1.0 d
	20	40 ± 6.0 d	78 ± 6.0 d	30 ± 4.0 d	5.5 ± 0.1 c	14 ± 2.0 e
	*p*-value	0.000 *	0.000 *	0.000 *	0.002 *	0.000 *

G%—Germination percentage; GI—Germination Index; CVG—Coefficient of Velocity of Germination; MGT—Mean Germination Time Values. Values are mean ± standard deviation; asterisk and different letters indicate statistically significant differences at *p*-value ≤ 0.05 among treatments in each species. *p*-value from ANOVA test.

**Table 5:** Phytotoxic effects of different concentrations of EOs (liquid phase) on two target species in post-emergence conditions.

Target Species	EO (μL/mL)	Damaged Seedlings (%)	Damaged Leaves (%)	Damaged Leaf Surface (%)	Phytotoxicity (0–10)
	***A. alba***				
*L. multiflorum*	0	0.0 ± 0.0 a	0.0 ± 0.0 a	0.0 ± 0.0 a	0 ± 0.0 a
	10	23.3 ± 3.0 b	17.8 ± 2.2 b	14.8 ± 2.0 b	2 ± 0.0 b
	20	42.5 ± 5.6 c	40.7 ± 2.2 c	33.1 ± 1.9 c	4 ± 1.0 c
	50	100.0 ± 0.0 d	93.3 ± 0.0 c	68.8 ± 7.1 d	6 ± 2.8 d
	100	100.0 ± 0.0 d	96.0 ± 3.8 c	74.4 ± 8.4 d	8 ± 2.1 d
	*p*-value	0.000 *	0.000 *	0.000 *	0.000 *
*S. alba*	0	0.0 ± 0.0 a	0.0 ± 0.0 a	0.0 ± 0.0 a	0 ± 0.0 a
	10	26.7 ± 3.9 b	22.2 ± 3.0 b	20.6 ± 2.5 b	2 ± 0.8 b
	20	69.4 ± 7.8 c	60.5 ± 6.3 c	52.2 ± 3.0 c	6 ± 1.1 c
	50	100.0 ± 0.0 d	100.0 ± 0.0 d	85.6 ± 5.9 d	9 ± 1.3 d
	100	100.0 ± 0.0 d	100.0 ± 0.0 d	98.4 ± 7.6 e	10 ± 1.2 d
	*p*-value	0.000 *	0.000 *	0.000 *	0.000 *
	***P. abies***				
*L. multiflorum*	0	0.0 ± 0.0 a	0.0 ± 0.0 a	0.0 ± 0.0 a	0 ± 0.0 a
	10	17.8 ± 2.0 b	15.6 ± 2.4 b	10.0 ± 2.8 b	1 ± 0.4 b
	20	42.7 ± 6.8 c	33.6 ± 3.2 c	25.9 ± 4.4 c	3 ± 1.0 c
	50	100 ± 0.0 d	96.7 ± 0.0 d	54.7 ± 6.3 d	5 ± 0.5 d
	100	100 ± 0.0 d	97.0 ± 3.3 d	76.2 ± 6.9 e	7 ± 1.4 e
	*p*-value	0.000 *	0.000 *	0.000 *	0.000 *
*S. alba*	0	0.0 ± 0.0 a	0.0 ± 0.0 a	0.0 ± 0.0 a	0 ± 0.0 a
	10	13.3 ± 3.2 b	12.2 ± 1.0 b	12.5 ± 2.2 b	1 ± 1.0 b
	20	51.1 ± 8.8 c	44.4 ± 6.7 c	27.7 ± 4.3 c	3 ± 0.6 c
	50	100 ± 0.0 d	83.3 ± 5.0 d	48.8 ± 5.3 d	5 ± 0.8 d
	100	100 ± 0.0 d	99.0 ± 1.9 e	67.2 ± 1.6 e	7 ± 2.6 e
	*p*-value	0.000 *	0.000 *	0.000 *	0.000 *
	***P. cembra***				
*L. multiflorum*	0	0.0 ± 0.0 a	0.0 ± 0.0 a	0.0 ± 0.0 a	0 ± 0.0 a
	10	28.9 ± 6.5 b	18.9 ± 3.5 b	13.3 ± 4.7 b	1 ± 0.4 b
	20	46.7 ± 6.6 c	41.1 ± 5.1 c	24.8 ± 4.1 c	2 ± 0.9 c
	50	86.7 ± 8.0 d	76.7 ± 9.0 d	59.1 ± 6.7 d	3 ± 1.3 c
	100	100.0 ± 0.0 e	91.0 ± 5.1 e	66.1 ± 12.7 d	6 ± 1.8 d
	*p*-value	0.000 *	0.000 *	0.000 *	0.000 *
*S. alba*	0	0.0 ± 0.0 a	0.0 ± 0.0 a	0.0 ± 0.0 a	0 ± 0.0 a
	10	28.9 ± 5.4 b	21.1 ± 5.7 b	18.5 ± 6.2 b	2 ± 0.7 b
	20	55.6 ± 5.8 c	54.4 ± 7.5 c	30.5 ± 4.0 c	3 ± 0.5 b
	50	100.0 ± 0.0 d	83.3 ± 4.8 d	52.0 ± 6.8 d	5 ± 1.1 c
	100	100.0 ± 0.0 d	97.0 ± 5.8 e	70.6 ± 12.7 e	7 ± 1.6 d
	*p*-value	0.000 *	0.000 *	0.000 *	0.000 *
	***P. mugo***				
*L. multiflorum*	0	0.0 ± 0.0 a	0.0 ± 0.0 a	0.0 ± 0.0 a	0 ± 0.0 a
	10	20.0 ± 4.1 b	18.9 ± 2.9 b	14.9 ± 3.5 b	1 ± 0.0 b
	20	55.6 ± 7.8 c	41.1 ± 6.9 c	32.7 ± 8.9 c	3 ± 1.0 c
	50	100.0 ± 0.0 d	86.7 ± 0.0 d	58.8 ± 9.8 d	5 ± 2.2 d
	100	100.0 ± 0.0 e	100.0 ± 0.0 e	80.7 ± 12.8 e	8 ± 1.1 e
	*p*-value	0.000 *	0.000 *	0.000 *	0.000 *
*S. alba*	0	0.0 ± 0.0 a	0.0 ± 0.0 a	0.0 ± 0.0 a	0 ± 0.0 a
	10	26.7 ± 7.0 b	20.0 ± 6.1 b	18.6 ± 5.6 b	2 ± 1.0 b
	20	57.7 ± 8.5 c	47.7 ± 5.5 c	39.5 ± 9.5 c	4 ± 1.0 c
	50	75.5 ± 6.7 d	71.1 ± 9.3 d	60.5 ± 8.4 d	7 ± 1.2 d
	100	100.0 ± 0.0 e	100.0 ± 0.0 e	86.3 ± 8.8 e	9 ± 0.9 e
	*p*-value	0.000 *	0.000 *	0.000 *	0.000 *

Values are mean ± standard deviation; asterisk and different letters indicate statistically significant differences at *p*-value ≤ 0.05 among treatments in each species. *p*-value from ANOVA test.

**Table 6:** Visual phytotoxicity rating scale.

Rating	Target Species Responses/Injury	Description
0	0	no symptoms
1	1–10	very slight symptoms
2	11–20	more severe, but not lasting symptoms
3	21–30	moderate and more lasting symptoms
4	31–40	medium and lasting symptoms
5	41–50	moderately heavy symptoms
6	51–60	heavy symptoms
7	61–70	very heavy symptoms
8	71–80	nearly destroyed leaves/seedlings
9	81–90	destroyed leaves/seedlings
10	91–100	completely destroyed leaves/seedlings

## Data Availability

All generated data are included in this article.
